# Hygienic Education

**Published:** 1904-07-23

**Authors:** 


### The Hospital.

A Journal of

The Medical Sciences and Hospital Administration.

Vol. XXXVI.—No. 930. JULY 23, 1904.

### Hygienic Education.

The deputation received by the President of the Education Board (Lord Londonderry) on Monday, the 11th instant, and the petition which it was the object of that deputation to present and to support, may be held to indicate the introduction of an entirely new and very important element into the whole subject of national education. The petition, which was signed by 14,608 medical practitioners whose signatures were decipherable and by 110 more whose signatures were undecipherable, set forth that the whole of the signatories, "having constantly before them the serious physical and moral conditions of degeneracy and disease resulting from the neglect and infraction of the elementary laws of hygiene, venture to urge the Central Education Authorities of the kingdom to consider whether it would not be possible to include in the curricula of the public elementary schools and to encourage in the secondary schools such teaching as may, without developing any tendency to dwell on what is unwholesome, lead all the children to appreciate at their true value healthful bodily conditions as regards cleanliness, pure air, food, drink, etc.," and, having directed the attention of the board to what has been done or is doing in this direction in the Army schools and in some colonies and foreign countries, proceeded to argue that the indicated instruction is especially needed with reference to the effects of alcohol. In the words of the petition itself, the signatories, "having regard to the fact that much of the degeneracy, disease, and accident with which medical men are called upon to deal is directly or indirectly due to the use of alcohol, and that a widespread ignorance prevails concerning not only the nature and properties of this substance, but also its effects on the body and the mind, would urge upon the educational authorities of the United Kingdom to include, in the simple hygienic teaching which they desire, elementary instruction at an early age on the nature and effects of alcohol." The signatories admit that a certain amount of hygienic instruction is already given by means of the Optional Code, but they allege that this is only received by a few pupils, and they urge that the instruction should be compulsory, and that it should be given at a much earlier age than at present.

We need hardly say either that we are heartily in accord with the petitioners or that we regard them as having come forward at a somewhat critical time to fulfil a public duty of the highest importance to the country and to all future generations of its inhabitants. The statements and arguments contained in the petition were powerfully pressed upon Lord Londonderry by Dr. Farquharson, M.P., whose long experience as physician to Rugby School as well as the interest which he has displayed in the House of Commons on the general question of physical degeneracy entitle his opinions to much respect, and he was well seconded or supported by Sir William Broadbent, Dr. Dryslwyn Griffiths, Mrs. Scharlieb, and other speakers. In point of argument, indeed, there can hardly be said to be two sides to the question; for it is common knowledge that the laws of health are either unknown to or neglected by the larger proportion of the population, whether gentle or simple. A great number of railway travellers, for example, do their best to bring the carriages which they occupy into line with the Black Hole of Calcutta, and when they have succeeded, and when the illness which they have striven after and earned duly visits them next day, they innocently wonder how and where they have "taken cold." They are quite sure that they kept the windows shut all the way, notwithstanding the complaints of a tiresome man who said he did not like to be suffocated. On questions of dietary at least an equal degree of ignorance prevails almost universally, insomuch that kind and hospitable ladies still urge wine or brandy upon their friends as something that will "strengthen them," or that will "keep the cold out," and straightway proceed to marvel that the comparatively poor are apt to carry out these views in a complete and practical manner. A very great deal has been done in this country in the course of the last half-century towards the attainment of what may be called public cleanliness. A few years ago, at all events, we were definitely in front of all other nations in this respect, and our diminished death-rates were abundant evidence of the soundness of the position which we had assumed. There is some reason to fear that we have been overtaken even in that department of sanitary reform, which we claimed as peculiarly our own; and it is certain that the industrial classes of other countries are now far in advance of us in the matter of cleanliness of person and of clothing. A visitor to France, or Belgium, or Germany cannot fail to be struck by the personal neatness and tidiness of working men and their wives and children; and in America a still higher standard prevails than on the Continent. An American manufacturer, as a rule, is compelled to provide his skilled workmen with lockers for their clothes and with facilities for washing. The men keep their working clothes at the factory, and change and wash before they leave it to go into the street or to travel by any public conveyance. The greasy and grimy persons constantly to be encountered in the tramcars of the London County Council are unknown in New York or Philadelphia; and in these cities quite well-dressed people can travel in the street cars without fear of having their clothing irreparably soiled by that of their neighbours. However, nothing is born full-grown, and even the teaching of hygiene must have a commencement. We heartily congratulate the framers of the petition upon the wisdom and boldness which they have displayed, and we wish all possible success to their meritorious endeavour.

